# Immune cell contribution to vascular complications in diabetes

**DOI:** 10.3389/fendo.2025.1549945

**Published:** 2025-05-21

**Authors:** Lingli Ma, Xuejiao Zhang, Zimeng Li, Qing Wang

**Affiliations:** Department of Endocrinology and Metabolism, China-Japan Union Hospital of Jilin University, Changchun, China

**Keywords:** diabetes, immune cells, vascular complications, inflammatory mechanisms, treatment strategies

## Abstract

Diabetes mellitus often results in vascular complications, significantly impacting patients’ well-being. This review focuses on the role of immune cells in these complications, examining their mechanisms, biomarkers, and treatment strategies. Immune cells, including macrophages, T cells, and B cells, contribute to the development of both macrovascular and microvascular complications by secreting inflammatory factors and modulating immune responses. For instance, in diabetic coronary artery disease, macrophages form foam cells and promote inflammation, whereas in diabetic nephropathy, an imbalance in T-cell subsets exacerbates the condition. Novel immune-related biomarkers, such as soluble cytokine receptors and specific microRNAs, offer new avenues for early diagnosis and monitoring. Current treatments focus on inflammation and oxidative stress, while emerging therapies, including stem cell treatment and precision medicine, show promise but also present challenges. This review systematically summarizes and analyzes pertinent research. Its significance lies in synthesizing current research findings, identifying knowledge gaps, and providing guidance for future basic research and clinical practice. By elucidating the critical role of immune cells in diabetic vascular complications, it aids in the development of new therapeutic targets and more effective treatment strategies. Moreover, the exploration of novel biomarkers opens up the possibility of early disease intervention, and the review of the current treatment landscape and challenges encourages clinicians to make more rational treatment decisions. Overall, the aim is to enhance patients’ prognoses, alleviate the medical burden, and advance progress in diabetes treatment.

## 1 Introduction

Diabetes, a chronic metabolic disease, includes type 1, type 2, and gestational diabetes, each with its own unique pathogenesis. It is associated with metabolic disorders and can lead to serious complications, such as cardiovascular and cerebrovascular diseases, severely impacting patients’ health and quality of life. According to the International Diabetes Federation (IDF) (1), the current global prevalence of adult diabetes is 9%, with one in eleven people being diabetic. Projections indicate that by 2045, the prevalence will reach 12.2%, showing a significant rise in the number of patients, high-risk individuals, and undiagnosed cases compared to the eighth-edition report (2). Both genetic and environmental factors, including the intrauterine environment, diet, obesity, and age, contribute to the development of diabetes. Furthermore, immune-mediated chronic inflammation plays a role in diabetic vascular complications (3). Considering the diversity of diabetic complications and patients’ responses to treatments, personalized medicine approaches that are based on immune profiles are becoming a significant frontier in clinical management. These approaches aim to customize therapeutic strategies to individual patients’ immune characteristics, thereby improving treatment efficacy and reducing adverse effects.

For instance, the collaboration between innate and adaptive immune cells in affecting the progression of diabetes, as well as the changes in the functions and relationships of immune cells in the early and late stages of the disease, are questions that require further investigation. On the other hand, most existing studies are based on animal experiments and *in-vitro* research, and there are still significant challenges in clinical translation. For instance, the efficacy and safety of treatment methods targeting immune cell targets in clinical trials still need to be further verified. This review aims to systematically integrate existing research findings, deeply analyze various aspects of the immune-diabetes relationship, including the detailed roles of immune cells in the pathogenesis of diabetes and the complex interactions between immune responses and metabolic disorders. Through a comprehensive review of relevant research, we can identify current research gaps and provide more targeted directions for subsequent basic research and clinical practice, thereby promoting the further development of the field of diabetes prevention and treatment.

## 2 Pathogenesis of diabetic vascular complications and immune system interactions

### 2.1 Pathogenic mechanisms of diabetic vascular complications

#### 2.1.1 Genetic susceptibility and metabolic disorders

Diabetic vascular complications can be categorized into macrovascular and microvascular complications, and their pathogenesis is highly complex, with no unified conclusion yet. Traditionally, genetic susceptibility is considered to play a significant role (4). Metabolism-related genes, such as the apolipoprotein B gene and lipoprotein lipase gene (5), as well as hemodynamics-related genes like the angiotensin-converting enzyme gene and endothelial nitric oxide synthase gene, are closely associated with diabetic vascular complications (6, 7). These genes play a role in the onset and progression of diabetic macroangiopathy by affecting the levels of body-expressed products. Variations in these genes can alter their expression or activity, causing dysfunctions that promote the development of atherosclerosis and ultimately lead to diabetic cardiovascular complications. This also offers potential genetic targets for personalized medicine and targeted therapies.

#### 2.1.2 Vascular endothelial dysfunction and oxidative stress

Moreover, metabolic disorders, vascular endothelial dysfunction, inflammatory factors, and oxidative stress are crucial in the development of diabetic vascular complications (8, 9). Metabolic disorders, including insulin resistance, hyperglycemia, hyperlipidemia, and obesity, are closely linked to diabetic vascular problems (10). The progression of insulin resistance parallels that of endothelial dysfunction in atherosclerosis (11). Hyperinsulinemia can thicken the blood vessel wall and promote lipid synthesis, exacerbating atherosclerosis (12). Vascular endothelial cell dysfunction, an early change in diabetic vascular disease, not only triggers atherosclerosis but also secretes biological factors and inflammatory proteins that further promote its development. Inflammatory factors, such as interleukins and tumor necrosis factor-α, significantly affect the development of diabetic angiopathy (13, 14). Oxidative stress, which arises from the accumulation of oxygen free radicals due to hyperglycemia, is associated with both macroangiopathy and is the primary cause of microvascular pathology (15). Immune cells and the inflammatory responses they initiate are implicated in numerous aforementioned pathogenic mechanisms. Consequently, conducting in-depth research on the role of immunity in diabetic vascular complications is crucial for uncovering their pathogenesis and for the development of new treatment strategies.

#### 2.1.3 Inflammatory network activation

Recent studies have highlighted the close association between immune responses and the pathogenesis of diabetes. Chronic overnutrition overactivates the immune system, disrupts immune cell functions, triggers obesity, and eventually leads to insulin resistance (16). This vicious cycle exacerbates metabolic disorders and significantly increases the risk of diabetes. Exploring the immune-diabetes relationship not only provides new insights into pathogenesis but also offers potential clues for developing effective therapies, which is crucial for improving the health of the growing diabetic population worldwide.

### 2.2 Bidirectional relationship between immunity and diabetes

#### 2.2.1 Immune-mediated pathogenesis in type 1 vs. type 2 diabetes

Type 1 diabetes (T1D) and type 2 diabetes (T2D) significantly differ in their impact on the immune response and the development of vascular complications. Regarding the immune response, T1D is an autoimmune disease where the immune system erroneously targets pancreatic islet β-cells (17), leading to a complete lack of insulin production. In this process, T lymphocytes, particularly CD4+ and CD8+ T cells, are pivotal. CD4+ T cells can differentiate into Th1 cells, which secrete pro-inflammatory cytokines such as interferon-γ (IFN-γ), activating the immune system and enhancing the inflammatory response. CD8+ T cells directly kill islet β-cells, disrupting their insulin-secreting function (18). This autoimmune attack persists, maintaining the body in a state of long-term immune activation, continuously releasing inflammatory factors. These factors, in turn, affect the function of vascular endothelial cells, increase oxidative stress, and promote the development of vascular complications.

Conversely, the immune response in T2D is primarily associated with metabolic inflammation. Obesity and insulin resistance are key features of T2D (19). Prolonged consumption of high-calorie diets and the presence of obesity result in the accumulation of adipose tissue. Adipocytes undergo hypertrophy and secrete numerous inflammatory factors, including tumor necrosis factor-α (TNF-α) and interleukin-6 (IL-6). Inflammatory factors activate the immune system, triggering chronic low-grade inflammation. This prompts macrophages to infiltrate the inflammatory site and polarize into the M1 type, further releasing pro-inflammatory mediators and exacerbating the inflammatory response (20). Simultaneously, in patients with T2D, there is an imbalance in T-lymphocyte subsets (21), characterized by an increase in Th1 and Th17 cells and a decrease in Th2 and regulatory T cells (Tregs). This immune regulatory dysfunction contributes to the development of vascular complications.

#### 2.2.2 Metabolic-immune crosstalk: from insulin resistance to chronic inflammation

There exists a complex bidirectional relationship between metabolic dysregulation and immune dysfunction. Metabolic abnormalities, including hyperglycemia, insulin resistance, and dyslipidemia, can provoke inflammatory responses, resulting in immune cell dysfunction (22). For instance, prolonged hyperglycemia induces macrophages to polarize towards the pro-inflammatory M1 phenotype (23), secreting numerous pro-inflammatory cytokines such as TNF-α and interleukin-1β (IL-1β), thereby exacerbating the inflammatory response. Simultaneously, it suppresses the function of anti-inflammatory M2 macrophages, thereby impacting tissue repair. In the state of insulin resistance, adipose tissue secretes increased inflammatory factors, activating the immune system and further exacerbating metabolic disorders, creating a vicious cycle (24). Conversely, immune dysfunction also adversely affects metabolism (25). In T1D, the immune system attacks pancreatic islet β-cells, resulting in insufficient insulin secretion and abnormal blood glucose metabolism. In T2D, the abnormal activation of immune cells can interfere with the insulin signaling pathway, reducing insulin sensitivity and exacerbating insulin resistance.

Interventions aimed at metabolic dysregulation significantly affect immune function. Metformin serves as a prime example. It not only enhances blood glucose control but also possesses anti-inflammatory properties. Metformin stimulates adenosine monophosphate-activated protein kinase (AMPK), suppresses the activity of nuclear factor-κB (NF-κB), decreases the production of inflammatory cytokines, and modulates the function of immune cells (26). Furthermore, insulin therapy, while addressing hyperglycemia, may also regulate immune function by influencing insulin receptors on immune cells (27). Interventions aimed at immune dysfunction also influence metabolism. For instance, the use of immunosuppressants to suppress an overactive immune system can assist in protecting pancreatic islet β-cell function and enhancing insulin secretion in certain autoimmune diabetes-related diseases, thereby positively affecting blood glucose metabolism. However, immunosuppressants may also have side effects, such as an increased risk of infection, which can impact the overall metabolic state.

## 3 Classification and functional profiles of immune cells in diabetes

### 3.1 Inherent immune cells

#### 3.1.1 Macrophages

Monocytes encompass mononuclear cells found in peripheral blood and macrophages located in various body tissues and organs. Their undivided nuclei originate from hematopoietic stem cells in the bone marrow, playing a crucial role in numerous diseases (28). In addition to serving as the first line of defense against infections, tumor development, and autoimmune diseases, they are also vital in preventing chronic inflammation. For instance, in atherosclerosis, monocytes migrate to the arterial wall and differentiate into macrophages, which then consume lipids to form foam cells, thereby exacerbating the progression of the disease (29, 30).

Human peripheral blood mononuclear cells can be categorized into three primary subgroups based on various surface markers: classical, intermediate, and nonclassical (**Table 1**) (31–33). The classification of these subgroups is determined by the expression levels of chemokine receptors such as C-C chemokine receptor type 2 (CCR2) and C-X3-C motif chemokine receptor 1 (CX3CR1), as well as cluster of differentiation markers including CD14, CD16, and CD64. These subsets can be further distinguished by varying levels of human leukocyte antigen-DR (HLA-DR), C-C chemokine receptor type 5 (CCR5), tumor necrosis factor receptor 1 (TNFR1), and tumor necrosis factor receptor 2 (TNFR2). Notably, intermediate monocytes exhibit the highest expression of TNFR1, whereas atypical monocytes display the highest expression of TNFR2.

**Table 1:** Human monocyte markers.

Group	Marker	Chemokine receptor	Function
Classic type 87-88%	CD14、CD64、 CD62L、TNFR1 TNFR2	CD192、CXCR1	Major groups with phagocytic activity Weak inflammatory cytokine production
Intermediate type (classic CD16+) 2-3%	CD16、CD14、 CD64、HLA-DR、 TNFR1、TNFR2	CD192、CX3CR1、 CD195	Promote inflammation Promote production of TNF-α, IL1b and IL-6. Resist inflammation
Nonclassical type 10%	CD14、CD16、 TNFR1、TNFR2	CX3CR1	Production of IL-1RA

Macrophages primarily originate from monocytes. Upon their release from the bone marrow, monocytes circulate throughout the body and settle in most tissues, where they mature into macrophages. They differentiate into various forms in different organs to adapt to the specialized functions therein, including osteoclasts, brain microglia, alveolar macrophages, peritoneal macrophages, liver Kupffer cells, and adipose tissue macrophages (34). A small fraction originates from embryonic cells, and these cells persist throughout life via self-renewal. Macrophages are effective immune effector cells, playing a crucial role in maintaining tissue homeostasis and facilitating tissue repair, including promoting wound healing and tissue regeneration in various pathological conditions (35).

Heterogeneity of monocytes in human peripheral blood. The figure illustrates the classification of monocytes into three main subgroups: classical, intermediate, and non-classical, based on surface markers and chemokine receptor expressions. Classical monocytes are characterized by high phagocytic activity, while intermediate monocytes show moderate cytokine production. Non-classical monocytes are involved in inflammatory responses, promoting TNF-α, IL-1β, and IL-6 production. The polarization of monocytes into these subsets plays a crucial role in immune responses, particularly in the context of diabetes and its vascular complications.

Macrophages are professional phagocytes that patrol most tissues, aiding in the maintenance of internal balance and serving as the first line of defense against pathogens. They are characterized by their high plasticity, enabling them to alter their phenotype in response to various environmental cues. Different phenotypes exhibit distinct characteristics and functions, playing regulatory roles in the body’s physiological and pathological processes, a phenomenon known as the polarization effect of macrophages (36). When stimulated by pathogenic microorganisms, inflammatory reactions, cytokines, or physical and chemical factors, macrophages undergo polarization. They differentiate into two main phenotypes based on the changes in their microenvironment: the alternatively activated M2 type and the classically activated M1 type (37).

M1 macrophages produce reactive oxygen species (ROS) by activating their oxidase system, which is involved in the elimination of pathogens during infection. Consequently, M1 macrophages primarily function in antigen presentation and promote inflammation, while also eliminating pathogenic microorganisms and resisting tumors (38). However, they also produce ROS-induced tissue damage, which can impair tissue regeneration and wound healing. To prevent such injury, the anti-inflammatory regulation of the polarized M2 macrophage is necessary (39). M2 macrophages exhibit their anti-inflammatory cytokine characteristics by secreting low levels of IL-12, high levels of IL-10, and transforming Growth Factor-β (TGF-β). They possess robust phagocytic capabilities, enabling them to clear debris and apoptotic cells (40). Compared to M1 macrophages, M2 cells are capable of promoting angiogenesis, fibrosis, tissue repair, and wound healing. Consequently, it is generally understood that M2 cells can suppress inflammation, facilitate tissue remodeling, angiogenesis, immune regulation, and contribute to the formation and progression of tumors (41).

#### 3.1.2 NK cell

Natural killer (NK) cells are lymphocytes that originate from the bone marrow and are classified as innate lymphocytes. The recognition receptors of NK cells consist primarily of two types: the immunoglobulin superfamily and the C-type lectin superfamily, each of which includes inhibitory and activating receptors (42). NK cells are not restricted by the major histocompatibility complex (MHC) when killing target cells; they identify virus-infected and mutant cells through a process known as “missing-self” recognition (43). Normal somatic cells display MHCI molecules on their surface, which can be detected by NK cells’ inhibitory receptors. This recognition triggers the inhibition of the cytotoxic activity of NK cells, thereby preventing them from attacking normal somatic cells. When somatic cells are infected by a virus or undergo a gene mutation, the expression of MHCI molecules on the cell surface is down-regulated or deleted, causing the cytotoxic inhibition mediated by inhibitory receptors to cease. Consequently, NK cells can initiate the destruction of target cells.

#### 3.1.3 Others

Neutrophils, also known as polymorphonuclear granulocytes, possess a lobulated nucleus and originate from bone marrow hematopoietic stem cells, constituting approximately 60% to 70% of all adult peripheral blood white cells (44). These cells can adhere to the surface of vascular endothelial cells and migrate through the gaps between them to reach the tissue sites where pathogenic microorganisms have infiltrated. Neutrophils are involved in acute inflammatory reactions, performing phagocytosis and bactericidal functions, which can be further amplified by the presence of antibodies and complements.

Eosinophils, which originate from the bone marrow, are rich in enzymes such as peroxidase and acid phosphatase. Their numbers in tissues are significantly higher than in peripheral blood (45), and they are primarily distributed in the respiratory tract, digestive tract, and urogenital mucosa. Eosinophils possess a certain phagocytic ability, enabling them to engulf and digest microorganisms, a process that is enhanced by the action of complement and antibodies.

Basophils originate from the bone marrow, which is the source of the fewest white blood cells in the peripheral blood of healthy individuals. The Fc receptor (FcϵR) expresses complement receptors and IgE on the surface of basophilic cells. The granules within basophils contain various bioactive mediators that can mediate the occurrence and progression of type I hypersensitivity (46).

**Figure 1:**
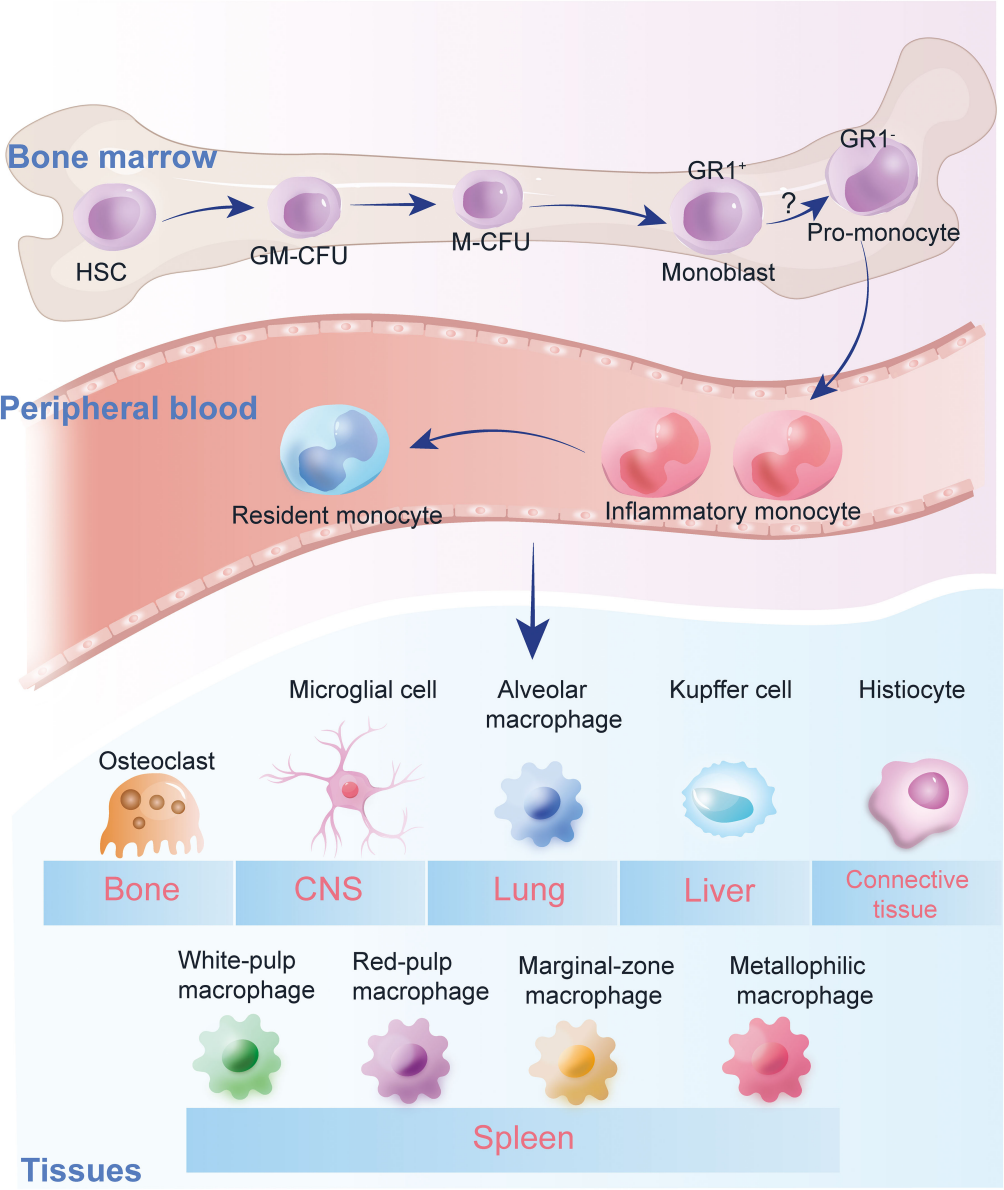
Monocyte heterogeneity. Heterogeneity of monocytes in human peripheral blood. The figure illustrates the classification of monocytes into three main subgroups: classical, intermediate, and non-classical, based on surface markers and chemokine receptor expressions. Classical monocytes are characterized by high phagocytic activity, while intermediate monocytes show moderate cytokine production. Non-classical monocytes are involved in inflammatory responses, promoting TNF-α, IL-1β, and IL-6 production. The polarization of monocytes into these subsets plays a crucial role in immune responses, particularly in the context of diabetes and its vascular complications.

Antigen-presenting cells (APC) are responsible for presenting antigens, which involves taking in microorganisms or other antigens, processing them, and presenting them to T lymphocytes (47). They also provide the necessary stimulating signals for T cell activation. Dendritic cells (DC), the primary type of APC that initiates the T cell immune response, are distributed beneath the epithelium and in various organs. They are capable of capturing antigens promptly and transporting them to peripheral lymphoid organs (48). The majority of DCs, also known as myeloid DCs, originate from monocytes. (**Figure 1**) Moreover, monocytes/macrophages possess a diverse array of receptors, which are activated by pathogens, immune complexes, or IFN-γ, and they internalize antigens via pinocytosis, regulatory phagocytosis, and receptor-mediated endocytosis (49). Through the expression of MHC class II/I molecules, they engage in the processing and presentation of both exogenous and endogenous antigens. Additionally, they highly express CD80/CD86 (B7-1/B7-2) and other molecules (50). The expression of high levels of co-stimulatory molecules, such as CD80/CD86, by these APCs is crucial for effective T-cell priming and activation. This ensures robust and appropriate immune responses against encountered pathogens or altered self-components. Therefore, the interplay between DCs, monocytes, and macrophages, along with their specialized functions in antigen presentation and T-cell activation, forms the foundation of adaptive immunity’s ability to recognize and eliminate a wide range of threats while maintaining self-tolerance (51).

### 3.2 Adaptive immune cells

#### 3.2.1 T cells

**Figure 2:**
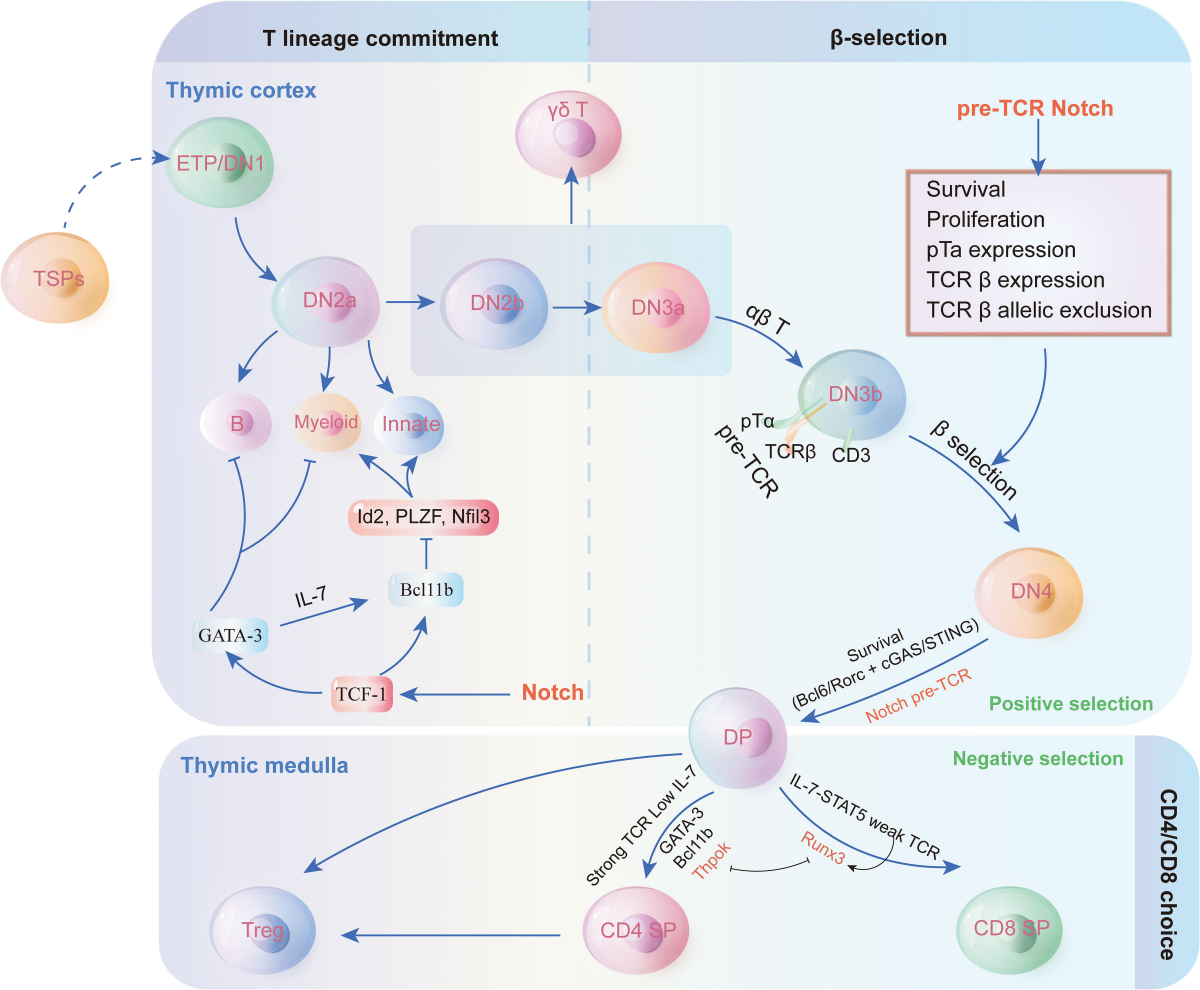
Thymus cell development and regulation mechanism. Thymocyte development and T cell lineage commitment in the thymus. The figure illustrates the progression of thymocyte differentiation from early thymic progenitors (ETP/DN1) through distinct double-negative (DN) stages to double-positive (DP) thymocytes. The β-selection checkpoint marks the transition from DN3 to DN4, driven by pre-TCR signaling and Notch activation, ensuring T cell survival, proliferation, and allelic exclusion of TCR β-chain. Post β-selection, thymocytes undergo positive and negative selection in the thymic cortex and medulla, leading to lineage commitment into CD4+ or CD8+ single-positive (SP) T cells. Additionally, the figure highlights the potential differentiation into γδ T cells, innate lymphoid cells, or other lineages under the influence of transcription factors like GATA-3, Id2, and Bcl11b. This pathway underscores the intricate regulatory mechanisms guiding T cell fate decisions within the thymic microenvironment.

T cells, also known as T lymphocytes, are a specialized category of white blood cells that are crucial for adaptive immunity (52). Adaptive immunity is a component of the immune system that evolves to recognize and remember specific pathogens. They collaborate with other immune cells, including B cells that generate antibodies to neutralize pathogens, and various white blood cells that ingest and eliminate foreign entities. The proper functioning of T cells is essential for the immune system to effectively combat infections and preserve overall health (53). Within the thymus, T cells primarily develop into CD4+ and CD8+ T cell subsets (54). (**Figure 2**) Naive T cells differentiate into CD4+ helper cells, CD8+ cytotoxic effector cells, and memory cells upon antigen stimulation, performing functions such as direct killing and immunomodulation, and offering long-term protection.

Thymocyte development and T cell lineage commitment in the thymus. The figure illustrates the progression of thymocyte differentiation from early thymic progenitors (ETP/DN1) through distinct double-negative (DN) stages to double-positive (DP) thymocytes. The β-selection checkpoint marks the transition from DN3 to DN4, driven by pre-TCR signaling and Notch activation, ensuring T cell survival, proliferation, and allelic exclusion of TCR β-chain. Post β-selection, thymocytes undergo positive and negative selection in the thymic cortex and medulla, leading to lineage commitment into CD4+ or CD8+ single-positive (SP) T cells. Additionally, the figure highlights the potential differentiation into γδ T cells, innate lymphoid cells, or other lineages under the influence of transcription factors like GATA-3, Id2, and Bcl11b. This pathway underscores the intricate regulatory mechanisms guiding T cell fate decisions within the thymic microenvironment.

Cytotoxic CD8+ T cells, also known as killer T cells, are responsible for directly attacking and destroying infected or abnormal cells. They recognize and bind to antigens (foreign substances) on the surface of infected cells, resulting in the destruction of target cells (55). Initially naive, CD8+ T cells require interaction with APCs to activate them and increase the upregulation of T cell Receptors (TCRs) specific to antigens. Following their migration into the bloodstream, the activated CD8+ T cells locate their antigen targets, which triggers the cells to undergo apoptosis. CD8+ T cells employ various methods to mediate their killing function. The first mechanism makes use of the Fas/Fas ligand (FasL) combination. FasL is expressed by activated CD8+ T cells, and when FasL binds to Fas expressed by some target cells, caspase activation occurs, which causes the target cells to undergo apoptosis (56). Granzyme and perforin release are involved in the second mechanism. Granzyme causes all target cell types to undergo apoptosis, and perforin creates holes in the target cell membrane so that granzyme can enter. TNF-α, tumor necrosis factor-related apoptosis-inducing ligand (TRAIL), and IFN-γ are among the cytokines secreted by activated CD8+ T cells, which mediate apoptosis through various signal transduction. Once the target cell undergoes apoptosis, it is usually swallowed and digested by other immune cells to remove cell debris (57–59).

Helper CD4+ T cells are pivotal in orchestrating the immune response. They assist in activating other immune cells, such as B cells and cytotoxic T cells, by secreting cytokines. CD4+ T cells become activated when MHCII molecules are presented on the surface of cells and antigens are displayed. Upon activation, they proliferate rapidly and secrete cytokines to regulate or sustain the immune response (60). These cells can differentiate into various subtypes, each with distinct functions.

#### 3.2.2 B cells

B cells originate from hematopoietic stem cells (HSC) and undergo a complex development process within the bone marrow (61). Lymphocytes initially differentiate into pre-B cells (Pre-B), which express the immunoglobulin α/β (Igα/Igβ) heterodimer, a key marker of B cells. The pre-B cell stage comprises two phases: early pre-B cells undergo heavy chain diversity-joining (D-J) gene segment recombination, whereas late pre-B cells experience variable-diversity-joining (V-DJ) gene segment recombination. Currently, when B cells start expressing the immunoglobulin heavy chain, they differentiate into Pre-B cells, which come in two types: large and small Pre-B cells (62). The small Pre-B cells do not produce a functional B cell receptor (BCR), but they initiate the recombination of the light chain V-J gene segment and synthesize a complete μ heavy chain, expressing the Pre-B receptor. Cells that have expressed surface immunoglobulin M (IgM) and completed the recombination of light chain V-J gene segments are referred to as immature B cells. Since many of these immature B cells are self-reactive, they must undergo negative selection to be eliminated. The B cells that successfully pass through these negative selection processes express surface immunoglobulin D (IgD) and mature into fully developed B cells. This series of complex developmental stages ensures that B cells in the immune system are both functional and self-tolerant.

#### 3.2.3 Dendritic cells

DC, a type of white blood cell found in mammals, functions as an APC. Its primary role is to present processed antigens to T cells of the immune system, acting as a bridge between adaptive and innate immunity (63). During inflammatory responses, DC cells process and present antigens, mature, and migrate to lymphoid tissues to present foreign substances to naïve T cells. Following activation, these early T cells differentiate into CD8+ T helper cells or cytotoxic CD8+ T cells. Even under non-inflammatory conditions, antigen presentation by DC cells is crucial. They induce the differentiation of regulatory T cells or the inactivation of autoreactive immune cells by presenting autoantigens or harmless environmental antigens (64).

DCs are the only APCs capable of activating initial T lymphocytes currently, and they are crucial for the development of atherosclerotic plaques (65). Elevated levels of advanced glycation end products (AGEs) in individuals with diabetes can exacerbate the inflammatory immune response of atherosclerosis by promoting the differentiation and maturation of DCs (66, 67).

## 4 Immune cell-mediated pathways in diabetic vascular complications

### 4.1 Diabetes macrovascular complications

#### 4.1.1 Diabetic coronary artery disease

As diabetes progresses, elevated glucose levels, advanced glycation end products, and oxidized low-density lipoprotein can lead to endothelial dysfunction (68). These factors promote the development of atherosclerosis by enhancing lipid deposition, inflammation, and oxidative stress in the coronary arteries. DCs are activated by the aforementioned factors, and their phenotypic maturation is accelerated. Activated CD4+CD28-Tregs are closely associated with the occurrence of diabetic macroangiopathy (69). CD4+CD28-Tregs can secrete IFN-γ, which stimulates macrophages to secrete inflammatory cytokines, causing damage to the vascular wall (70). Simultaneously, the cytotoxic perforin produced by CD4+CD28-Tregs can also directly damage endothelial cells (71). Currently, potential therapeutic approaches primarily focus on anti-inflammatory and immunomodulatory treatments. For instance, drugs that inhibit the activation of immune cells or the production of inflammatory cytokines may be effective in reducing the risk of diabetic coronary artery disease. However, further clinical trials are required to verify their long-term efficacy and safety.

This figure provides an overview of the mechanisms by which various immune cells act in diabetic vascular complications. In the macrovascular complication area on the left, immune cells including macrophages, T cells, and dendritic cells promote the development of diseases like diabetic coronary artery disease, cerebrovascular disease, and peripheral artery disease through different inflammatory pathways. In the microvascular complication area on the right, immune cells such as macrophages, T cells, B cells, and others contribute to the progression of diabetic nephropathy, diabetic foot, diabetic retinopathy, and diabetic peripheral neuropathy. The arrows and labels clearly illustrate the specific interactions between immune cells and the pathological processes of these complications, offering a comprehensive understanding of the immune-mediated mechanisms in diabetic vascular complications.

#### 4.1.2 Diabetic cerebrovascular disease

**Figure 3:**
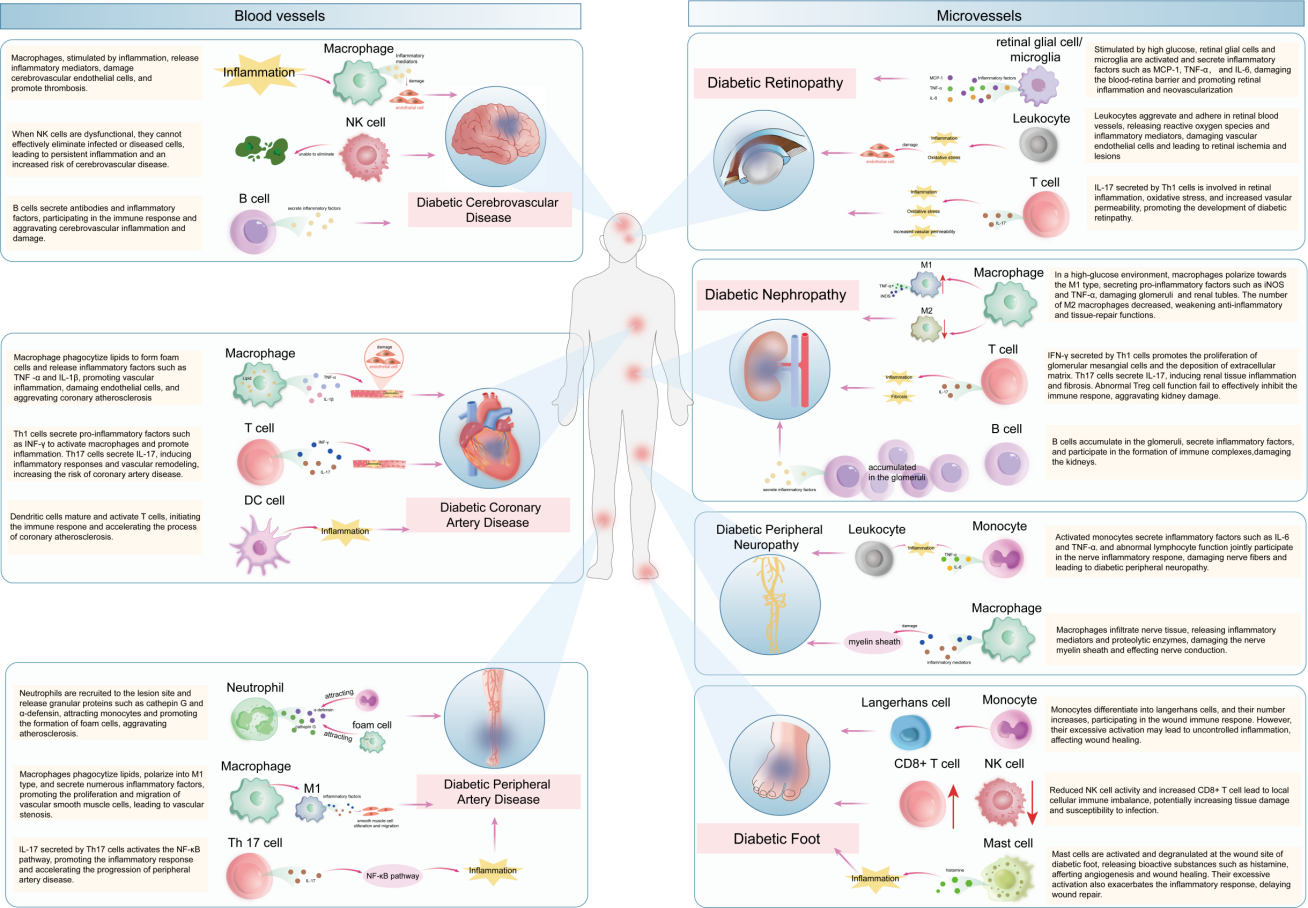
the mechanisms of immune cells in diabetic vascular complications. This figure provides an overview of the mechanisms by which various immune cells act in diabetic vascular complications. In the macrovascular complication area on the left, immune cells including macrophages, T cells, and dendritic cells promote the development of diseases like diabetic coronary artery disease, cerebrovascular disease, and peripheral artery disease through different inflammatory pathways. In the microvascular complication area on the right, immune cells such as macrophages, T cells, B cells, and others contribute to the progression of diabetic nephropathy, diabetic foot, diabetic retinopathy, and diabetic peripheral neuropathy. The arrows and labels clearly illustrate the specific interactions between immune cells and the pathological processes of these complications, offering a comprehensive understanding of the immune-mediated mechanisms in diabetic vascular complications.

Inflammatory factors produced by immune cells are crucial in the pathogenesis of diabetic cerebrovascular disease. These factors can cause insulin resistance, disrupt blood sugar regulation, and lead to inflammatory damage in blood vessels. They also promote platelet aggregation, accelerate thrombosis, and inhibit vascular endothelial function, thereby increasing vascular endothelial permeability (72). Consequently, blood lipids and other substances are more easily deposited in the damaged vascular endothelium, contributing to the thickening of the vessel lumen and the formation of atherosclerotic plaques. Monocytes and macrophages are recruited to the site of vascular injury (73). (**Figure 3**) Monocytes can differentiate into macrophages, which secrete a variety of inflammatory cytokines and proteases, further aggravating the inflammatory response and vascular damage. Additionally, an imbalance in T-cell subsets may also contribute to the development of diabetic cerebrovascular disease (74). For instance, an increase in Th1 and Th17 cells and a decrease in Tregs can lead to enhanced inflammation. The analysis of the relationship between inflammatory factors and cerebrovascular diseases revealed that the levels of monocytes, soluble vascular cell adhesion molecule-1, soluble intercellular adhesion molecule-1, C-reactive protein, and tumor necrosis factor-α chemoattractant protein in the healthy group, unincorporated group, and combined group exhibited a progressive increase. The differences between these groups were significant, indicating that the levels of inflammatory factors in patients’ bodies increased notably following the onset of cerebrovascular diseases, and there was a correlation between them (75). Treatment strategies for diabetic cerebrovascular disease often include anti-inflammatory drugs and medications that enhance vascular endothelial function (76). Some drugs can inhibit the production of inflammatory factors or regulate the immune response, aiming to reduce the risk of cerebrovascular events. However, the development of more targeted therapies, based on specific immune mechanisms, is still ongoing.

#### 4.1.3 Diabetic peripheral arterial disease

Atherosclerosis is a key pathological feature of diabetic peripheral arterial disease. Oxidative stress and inflammation, closely related to diabetes, interact to promote the occurrence and development of atherosclerosis (77). The imbalance of the gut microbiome in diabetic patients can also contribute to the disease process (78). When Gram-negative bacteria in the gut die, lipopolysaccharide (LPS) is released, activating macrophages and promoting inflammation at the lesion site, leading to vascular remodeling and plaque instability (79). Moreover, the gut microbiome can affect the production of short-chain fatty acids (SCFAs). In cases of diabetes, a reduction in beneficial bacteria that produce SCFAs, such as Bifidobacterium and Akkermansia muciniphila, is commonly observed (80). SCFAs are essential for maintaining intestinal homeostasis, regulating immune function, and decreasing inflammation. A decrease in their production can disrupt the intestinal barrier function, increase gut permeability, and allow endotoxins to enter the bloodstream, further aggravating systemic inflammation and facilitating the progression of diabetic peripheral arterial disease.

At the atherosclerotic lesion, activated platelets release chemokines that recruit and activate neutrophils. Neutrophils subsequently release granular proteins, such as cathepsin G and α-defensin, which attract monocytes to the lesion site (81). Monocytes differentiate into macrophages, and the resulting foam cells and activated macrophages further promote atherosclerosis. Additionally, Th17 cells can be activated by cytokines released from macrophages, enhancing the inflammatory response (82). The gut microbiome also significantly influences immune cells. Lipopolysaccharide (LPS), derived from Gram-negative bacteria in the gut, can activate macrophages through Toll-like Receptor 2/4 (TLR2/4) receptors (83). This activation results in the polarization of macrophages towards the M1 phenotype, enhancing the secretion of pro-inflammatory cytokines such as TNF-α, IL-1β, and IL-6. Additionally, the gut microbiome can influence the differentiation and function of T cells (84). For instance, it can alter the balance between Th17 cells and Tregs. An imbalance in the gut microbiome might cause an elevation in Th17 cells and a reduction in Tregs, which further disturbs the immune equilibrium and fosters chronic inflammation in diabetic individuals, thereby contributing to the advancement of peripheral arterial disease.

Current treatments for diabetic peripheral arterial disease include medications that improve blood circulation, such as vasodilators, and drugs that reduce inflammation (85). Emerging therapies target specific immune pathways, aiming to inhibit the activation of immune cells and the inflammatory response. Considering the role of the gut microbiome, probiotics and fecal microbiota transplantation (FMT) are being explored as potential treatment strategies. Probiotics, which contain beneficial bacteria, may help restore the balance of the gut microbiome, enhance intestinal barrier function, and reduce inflammation (86). FMT involves transferring the fecal microbiota from a healthy donor to a patient, which can potentially re-establish a healthy gut microbiome and improve the immune microenvironment (87). However, the long-term effects and safety of these treatments, particularly in relation to gut microbiome-related therapies, need to be further evaluated.

### 4.2 Diabetic microvascular complications

#### 4.2.1 Diabetic nephropathy

The pathogenesis of diabetic nephropathy (DN) is complex and involves multiple factors. Elevated glucose levels can activate various metabolic pathways, including the polyol pathway, the hexosamine pathway, and glycosylation reactions, leading to the production of numerous cytokines and growth factors (88). These substances can cause glomerular hypertrophy, thickening of the basement membrane, and expansion of the mesangial matrix, ultimately resulting in impaired renal function. Antigen-presenting DC are activated by antigens related to high glucose levels, linking innate and adaptive immune responses. In a non-immune-mediated residual kidney model, CD1a+CD80+ DCs accumulate in blood vessels, renal tubules, and the interstitium, particularly in the latter (89). The extent of DC aggregation is directly correlated with the development of tubulointerstitial fibrosis and the loss of renal function (90). Macrophage polarization is a significant factor in nephritis injury. M1 macrophages secrete a large number of pro-inflammatory cytokines, such as iNOS and TNF-α, which exacerbate inflammation and tissue damage. In contrast, M2 macrophages are involved in anti-inflammation and tissue repair, but their function is often compromised in diabetic nephropathy. In mice with DN and a macrophage-specific knockout of the cyclooxygenase-2 (COX2) gene, the number of M2 cells in the renal tissue decreased, proteinuria increased, and renal fibrosis exacerbated. The proportion of M2 cells was negatively correlated with the severity of DN symptoms (91). Pentraxin-3 (Ptx3), a member of the conserved protein superfamily, can assist DN patients with renal damage by promoting the differentiation of the M2 subgroup (92). T cells also play different roles. Th1 and Th17 cells promote inflammation, whereas Th2 cells have an anti-inflammatory effect. CD4+Foxp3+ Treg cells have a dual-edged effect, capable of regulating inflammation while also contributing to insulin resistance and the development of diabetic nephropathy. The kidney is harmed by both the indirect signaling pathway of cytokines and the direct cell-to-cell signaling pathway of surface molecules (93). Thus, by modulating the activity of CD8+ T cells, Tregs fulfill a dual function: they assist in controlling inflammation, yet simultaneously, they may inadvertently contribute to insulin resistance and the progression of DN. This underscores the fine balance within the immune system and its profound implications for metabolic and autoimmune disorders.

Research on the role of B cells in the pathophysiology of DN is currently limited. B cell clusters were observed in the glomeruli of non-obese diabetic mice, constituting about 1% of the renal lymphocytes in normal mouse kidneys—a relatively rare occurrence. In T1D mice, the number of glomerular B cells increased two to threefold, suggesting a potential involvement of B cells in the progression of diabetic neuropathic pain (94).

Drug treatments for diabetic nephropathy primarily target oxidative stress, inflammation, and the renin-angiotensin-aldosterone system (RAAS). For instance, antioxidants can mitigate oxidative stress, while RAAS inhibitors can decelerate the progression of renal damage (95). Innovative drugs, such as finerenone, a non-steroidal mineralocorticoid receptor antagonist, have demonstrated potential in safeguarding kidney function (96). Moreover, stem cell therapies, including the application of mesenchymal stem cells, are under investigation for their capacity to regenerate damaged renal tissues. However, their clinical use still encounters challenges like immune rejection and the risk of tumor formation.

#### 4.2.2 Diabetic foot

The development of diabetic foot ulcers is associated with multiple factors, including neuropathy, vasculopathy, and changes in the immune microenvironment. Neuropathy leads to loss of sensation and abnormal foot mechanics, increasing the risk of foot ulcers. Vasculopathy impairs blood circulation, affecting wound healing. The immune microenvironment in diabetic foot is altered, with an increase in the proportion of activated mast cells, monocytes, and CD8+ T cells, which may contribute to inflammation and tissue damage (97, 98).

Mast cells contribute to angiogenesis, inflammatory responses, and extracellular matrix reabsorption during wound healing. In cases of diabetic foot, the proportion of activated mast cells rises, potentially hindering the healing process (99). Shin et al. reported that the topical application of the mast cell degranulation inhibitor MCS-01 can accelerate wound healing in diabetic mice, whether applied before or after injury (100). Monocytes can differentiate into Langerhans cells in the spinous layer. The increased proportion of Langerhans cells may be involved in the immune response at the wound site. NK cells and CD8+ T cells are also involved in the local cellular immunity. The decrease in activated NK cells and the increase in CD8+ T cells may indicate an imbalance in local cellular immunity, but the specific mechanism requires further study.

Treatment of diabetic foot often involves wound care, infection control, and improvement of blood circulation. Some drugs that target immune cells, such as mast cell degranulation inhibitors, have shown potential in promoting wound healing. In addition, photobioregulation and conditioned medium have also been investigated for their effects on reducing inflammation and promoting wound repair.

#### 4.2.3 Diabetic retinopathy

The pathophysiology of diabetic retinopathy (DR) is characterized by inflammation and retinal neurodegeneration (101). Hyperglycemia can activate retinal glial cells and microglia, leading to the production of inflammatory cytokines such as monocyte chemotactic protein-1, vascular endothelial growth factor, TNF-α, and IL-6. These cytokines can disrupt the blood-retina barrier, promote angiogenesis, and cause retinal ischemia and neurodegeneration. Retinal glial cells and microglia are significant immune-related cells within the retina. Upon activation, they secrete inflammatory cytokines that facilitate the progression of diabetic retinopathy (102). Leukocytes, which include neutrophils and lymphocytes, also play a role. Chronic inflammation in diabetic patients can lead to leukocyte activation and increased infiltration into the retina, causing damage to retinal microcirculation. T cells, particularly Th17 cells, are capable of secreting IL-17, a cytokine implicated in retinal inflammation, oxidative stress, and increased capillary permeability (103). Current therapeutic strategies for diabetic retinopathy primarily concentrate on managing blood sugar, blood pressure, and lipid levels. Moreover, anti-vascular endothelial growth factor (VEGF) medications are extensively utilized to curb angiogenesis. Therapies targeting oxidative stress and inflammation, such as antioxidants and anti-TNF-α monoclonal antibodies, have also been investigated. Nonetheless, these treatments are not without their limitations, and there is a pressing need for more effective therapies that are grounded in a more profound comprehension of the immune mechanisms involved.

#### 4.2.4 Diabetic peripheral neuropathy

Diabetic peripheral neuropathy is caused by a combination of inflammatory responses and metabolic disorders. Inflammatory factors, such as C-reactive protein, are elevated in diabetic patients, and the activation of monocytes and lymphocytes is also observed. These factors can lead to nerve damage and affect nerve conduction. Monocytes and lymphocytes play significant roles in the inflammatory response associated with diabetic peripheral neuropathy (104). Activated monocytes secrete inflammatory cytokines that can damage nerve fibers. An imbalance in lymphocyte subsets, characterized by an increase in Th1 and Th17 cells and a decrease in Tregs, can lead to enhanced inflammation and immune-mediated nerve damage. Treatment for diabetic peripheral neuropathy typically involves controlling blood sugar levels and using medications to alleviate pain and enhance nerve function (105). Research is ongoing into drugs targeting inflammation, including anti-inflammatory agents and immune response regulators. Nonetheless, the development of more effective and targeted therapies continues to be a challenge.

## 5 Novel immune-related biomarkers for early diagnosis and prognosis

In recent years, the discovery and research of novel immune-related biomarkers have become a crucial area in the study of diabetic vascular complications. These biomarkers have the potential to serve as valuable tools for early detection, diagnosis, and monitoring of the progression of these complications.

### 5.1 Soluble cytokine receptors

Soluble cytokine receptors, such as the soluble tumor necrosis factor receptor (sTNFR), have shown promise as biomarkers for diabetic vascular complications. In cases of diabetes, the levels of sTNFR are often elevated, which correlates closely with the extent of inflammation and the severity of vascular damage (106). A prospective cohort study involving 607 patients with T2D found that elevated levels of sTNFR1 and sTNFR2 were significantly associated with increased risks of diabetic nephropathy, cardiovascular events, and all-cause mortality, independent of renal function and microalbuminuria (107). This suggests that sTNFR could potentially serve as a biomarker for predicting the development and progression of diabetic nephropathy, enabling earlier intervention and treatment (108).

### 5.2 Specific microRNAs

MicroRNAs (miRNAs) are small non-coding RNAs that play a significant role in regulating gene expression. In the context of diabetic vascular complications, specific miRNAs have been identified as potential biomarkers (109). One case-control study demonstrated significantly upregulated intravitreal miR-124 and miR-126-3p, downregulated miR-200b, and elevated VEGF levels in patients with proliferative diabetic retinopathy (PDR), suggesting these molecular alterations contribute to PDR pathogenesis and may serve as potential diagnostic or therapeutic targets (110). MiR-150 is downregulated in diabetes and DR, with its downstream targets being upregulated. These targets correlate with diabetes-associated inflammation, oxidative stress, apoptosis, and pathological angiogenesis. Consequently, MiR-150 may serve as a biomarker for predicting the occurrence of diabetic retinopathy and other vascular complications (111).

### 5.3 Circulating immune cell subsets

Certain circulating immune cell subsets can also serve as biomarkers for diabetic vascular complications. For instance, specific phenotypes of monocyte subsets have been linked to the progression of atherosclerosis in diabetes (112, 113). The proportion of classical monocytes expressing high levels of CD14 and CD64 may increase in patients with diabetic peripheral arterial disease. These monocytes are more prone to infiltrate the arterial wall, secrete inflammatory cytokines, and promote the development of atherosclerotic plaques. Studies have demonstrated that Th17 cells promote inflammatory cell infiltration, increase vascular permeability, and induce neovascularization by targeting retinal vascular endothelial cells and activating pro-inflammatory pathways (114). Their abnormal accumulation in retinal tissues correlates significantly with the severity of DR, and together with regulatory T cell dysfunction, constitutes a critical immune signature for DR progression (115). Monitoring the changes in the proportion of these monocyte subsets can provide insights into the status of vascular inflammation and the risk of diabetic vascular complications.

## 6 Immune-targeted therapies for diabetic vascular complications

### 6.1 Current pharmacological interventions

#### 6.1.1 Anti-inflammatory agents

As a frontline drug for type 2 diabetes, metformin inhibits nuclear NF-κB signaling by activating AMPK, thereby reducing the release of pro-inflammatory cytokines in macrophages and T cells (**Table 2**). In the context of macrovascular complications, it decreases the expression of vascular endothelial adhesion molecules, inhibiting monocyte infiltration into the vascular wall and slowing the progression of atherosclerotic plaques (116). Metformin has been shown to promote the polarization of macrophages towards the anti-inflammatory M2 phenotype, thereby alleviating renal interstitial fibrosis in DN and enhancing the blood-retina barrier (117, 118). This is achieved by inhibiting the activation of the NOD-Like receptor protein 3 (NLRP3) inflammasome in retinal microglia (119). Clinical studies have confirmed that long-term use of metformin reduces cardiovascular events in diabetic patients, a benefit that is partly attributed to its ability to inhibit the overactivation of innate immune cells.

**Table 2:** Drugs for inhibiting apoptosis signaling pathway in treating diabetic complications.

Drug name	Mechanism of inhibiting apoptosis	Complication	References
Metformin	NF-κB/TLR4 pathway, IGF-1, VEGF and TNF-α	DR	(120)
Mcc950	NLRP3 inflammasome, IL-1β	DR	(121)
Dapagliflozin	NLRP3、Caspase-1、IL-18、NF-κB	DR	(122)
NQO1	TLR4/NF-κB and TGF-β/Smad signaling pathways, IL-6、TNF-α、MCP-1	DN	(123)
Polyphenol extract with atorvastatin	AKT/mTOR/HIF-1 signaling pathway	DAS	(124)
TBK1 inhibitor amlexanox	NF-κb-related NLRP3 inflammasome activation and GSDMD-sparked microglia pyroptosis	DN	(125)
Baicalin	Nrf2 pathway and signaling pathway MAPK family, such as Erk1/2, JNK and P38, IL-1β 、IL-6、MCP-1 and TNFα	DN	(126)
San Huang Xiao Yan recipe	AMPK/Nrf2 pathway	DF	(127)
PEG-Loxe	GRP78/PERK/eIF2α pathway-related proteins, TLR4/NF-κB inflammatory pathway	DN	(128)

DR, Diabetic Retinopathy; DN, Diabetic Nephropathy; DAS, Diabetic Atherosclerosis; DF, Diabetic Foot; NF-κB, Nuclear Factor kappa B; TLR4, Toll-like Receptor 4; IGF-1, Insulin-like Growth Factor 1; VEGF, Vascular Endothelial Growth Factor; TNF-α, Tumor Necrosis Factor alpha; NLRP3, NOD-like Receptor Protein 3; Caspase-1, Cysteinyl Aspartate-Specific Proteinase 1; IL-18, Interleukin-18; NQO1, NAD(P)H:quinone oxidoreductase 1; TGF-β, Transforming Growth Factor beta; Smad, Smad proteins; IL-6, Interleukin-6; MCP-1, Monocyte Chemoattractant Protein-1; AKT, Protein Kinase B; mTOR, Mechanistic Target of Rapamycin; HIF-1, Hypoxia-Inducible Factor-1; TBK1, TANK-binding Kinase 1; GSDMD, Gasdermin D; Nrf2, Nuclear Factor Erythroid 2-related Factor 2; MAPK, Mitogen-Activated Protein Kinase; Erk1/2, Extracellular Signal-Regulated Kinases 1 and 2; JNK, c-Jun N-terminal Kinases; p38, p38 Mitogen-Activated Protein Kinase; AMPK, 5’ Adenosine Monophosphate-Activated Protein Kinase; GRP78, Glucose-Regulated Protein 78; PERK, Protein Kinase R-like Endoplasmic Reticulum Kinase; eIF2α, Eukaryotic Initiation Factor 2 alpha.

Beyond glucose reduction, sodium-glucose cotransporter 2 (SGLT2) inhibitors enhance the immune microenvironment through various mechanisms: ①they decrease renal medullary sodium load, thereby inhibiting RAAS-mediated T cell activation; ②they prevent the assembly of the NLRP3 inflammasome in macrophages, which reduces the release of IL-1β/IL-18; ③they promote the proliferation of Treg cells and inhibit the differentiation of pro-inflammatory Th17 cells (129, 130). Moreover, these agents decrease oxidative stress and neutrophil extracellular trap (NET) formation, which in turn lowers the thrombotic risk associated with diabetic cerebrovascular disease.

Glucagon-like peptide-1 (GLP-1) receptor agonists enhance immune balance by modulating T cell subsets: they promote the secretion of IL-10 by Tregs and inhibit the secretion of IFN-γ by Th1 cells and IL-17 by Th17 cells, thereby decreasing inflammation of the vascular wall (131). In cases of diabetic coronary artery disease (DCAD), treatment with semaglutide is associated with a reduced coronary fat attenuation index (FAI), indicating decreased macrophage infiltration and foam cell formation within plaques (132). Animal studies have revealed that GLP-1 signaling suppresses retinal microglial activation and VEGF-induced vascular leakage, suggesting potential protection against DR (133). Clinical evidence also shows that these agents lower the incidence of cardiovascular events in diabetic patients, with their immunomodulatory effects being closely associated with improved adipose tissue macrophage polarization (134).

#### 6.1.2 Immune modulators

Statins, by inhibiting the mevalonate pathway, not only lower cholesterol but also exert broad regulation on innate and adaptive immunity: ① they inhibit monocyte differentiation into pro-inflammatory M1 macrophages, thereby reducing foam cell formation in arterial walls; ② they decrease MHC-II expression on DCs, weakening antigen presentation and T cell activation; ③ they promote Treg proliferation and inhibit Th17-mediated glomerular inflammation (135, 136). Atorvastatin reduces podocyte pyroptosis and proteinuria in DN models by regulating the MALAT1/miR-200c pathway (137). (**Table 2**) Despite controversial effects on microvascular complications, statins are recommended as basic therapy for diabetic macrovascular disease, particularly in patients with hypercholesterolemia.

TNF-α is a key mediator that connects insulin resistance with vascular inflammation. Its inhibitors block the NF-κB pathway by neutralizing TNF-α, which reduces the release of pro-inflammatory cytokines from macrophages and endothelial cells. In DR, infliximab improves macular edema by inhibiting retinal vascular endothelial adhesion molecules and reducing leukostasis (138). (**Table 3**) However, its clinical use is limited due to the risk of infection and the potential for exacerbating heart failure, making them a second-line treatment option for refractory cases.

**Table 3:** Clinical trials summary of vascular complications associated with diabetes.

Disease	ClinicalTrials.gov ID	Compound	Target	Study Design	Clinical outcomes	Outcome Measure	limitations
DCAD	NCT01327846	Canakinumab	IL-1β	Randomized, double-blind, placebo-controlled phase III trial	150 mg dose: 15% reduction in primary endpoint	Primary: Nonfatal MI, stroke, cardiovascular death Secondary: Unstable angina requiring revascularization	Higher fatal infection rate
DCAD	N/A	Semaglutide	GLP-1 receptor	Retrospective cohort study	semaglutide treatment was correlated with a decreased pericoronary FAI of the LAD	Coronary inflammation (FAI of LAD/LCX/RCA)	Retrospective design without Randomization; Indirect inflammatory surrogate (FAI) without direct biomarker assessment; Limited sample size
DCAD	NCT01594333	Methotrexate	IL-1β、IL-6、CRP	Randomized, Double-Blind, Placebo-Controlled	No reduction in IL-1β, IL-6, or CRP levels; no decrease in cardiovascular event rates	Nonfatal MI, nonfatal stroke, cardiovascular death, hospitalization for unstable angina leading to urgent revascularization	Failed to validate hypothesized anti-inflammatory effect, short follow-up duration, and methotrexate-related adverse effects
DCD	NCT00722631	Pioglitazone	PPAR-γ	Prospective, randomized, comparator-controlled	Pioglitazone significantly reduced coronary artery inflammation	TBR, High-sensitivity hsCRP	Small sample size, short study duration (16 weeks), lack of long-term follow-up, patient dropouts
DN	NCT02358096	ASP8232	Vascular adhesion protein-1	Randomized, double-blind, placebo-controlled phase II trial	At 12 weeks, UACR decreased by 17.7% in the ASP8232 group	Mean change from baseline to week 12 in log-transformed first morning void UACR	Potential long-term safety issues are not clear
DN	NCT01447147	CCX140-B	CCR2	Randomized, double-blind, placebo-controlled phase II trial, multicenter	5 mg CCX140-B: 18% reduction in UACR from baseline (vs. Placebo -2%, p=0.01) 10 mg CCX140-B: 11% reduction in UACR from baseline (p=0.08)	Primary: Subject incidence of adverse events Secondary: Change from baseline in first morning urinary albumin:creatinine ratio (ACR)	10 mg dose did not reach statistical significance No assessment of hard endpoints (e.g., end-stage kidney disease, cardiovascular events)
DN	NCT01712061	CCR2/5 antagonist	PF-04634817	Randomized, double-blind, placebo-controlled phase II parallel-group	lacebo-adjusted 8.2% reduction in UACR at 12 weeks (95% credible interval: 0.75–1.09, not significant)	Primary: Change in UACR from baseline at 12 weeks	Clinical development discontinued due to modest efficacy
DN	NCT01648153	GSK1070806 Monoclonal antibody	Anti-Human IL-18	Multi-center, randomized, single-blind (sponsor-unblinded), placebo-controlled, parallel-group, Phase IIa clinical trial	No statistically significant effects of GSK1070806 on fasting plasma glucose levels and weighted mean glucose AUC compared to placebo.	Fasting plasma glucose, weighted mean glucose AUC over 4 hours after a mixed meal test	Although the drug was well-tolerated, the study limitations may preclude the exclusion of smaller, potentially clinically meaningful effects of IL-18 inhibition.
DN	NCT01774981	LY3016859 Monoclonal antibody	Anti-Human TGFα/Epiregulin	Randomized, double-blind, placebo-controlled, single and multiple ascending dose studies	LY3016859 was well-tolerated, but no significant reduction in proteinuria or albuminuria was observed in patients with diabetic nephropathy	Change From Baseline in Proteinuria	Small sample size; short study duration (16 weeks) relative to renal function assessments; proteinuria as a surrogate marker has limitations; study design did not assess disease progression delay
DR	NCT00505947	Infliximab	TGFα	Single-center, double-blind, randomized, placebo-controlled, crossover trial	Infliximab improved BCVA by 24.3% vs. placebo	Primary: Percentage difference in BCVA using mixed-models analysis for imbalanced crossover design	Small sample size (11 patients) Single-center design Short follow-up (32 weeks) Crossover design may introduce carryover effects No assessment of long-term safety or structural outcomes (e.g., retinal thickness)
DR	NCT00505947	Lifitegrast (SAR 1118)	LFA-1	Randomized, Double-Masked, Placebo-Controlled, Cross-Over, 32 Weeks Study	Terminated	improvement in best corrected visual acuity	The number of the anticipated participants was not achieved
DR	NCT02348918	Luminate^®^ (Alg-1001)	Integrins	Phase II Multi center, Randomized, Controlled, Double-Masked Clinical Trial	Non-inferiority to bevacizumab in BCVA and CMT for the treatment of DME	Change in BCVA at Week 24	Short follow-up
DR	NCT00511875	Doxycycline monohydrate	microglial	Randomized, double-masked, 24-month proof-of-concept clinical trial.	Mean FDP foveal sensitivity increased in the doxycycline group and decreased in the placebo group	FDP foveal sensitivity.	Small sample size No significant difference in anatomic outcomes

DCAD, Diabetic coronary artery disease; NCT, National Clinical Trial; N/A, Not Applicable; IL - 1β, Interleukin - 1 beta; MI, Myocardial Infarction; GLP - 1, Glucagon - like peptide - 1; FAI, Fat attenuation index; LAD, Left Anterior Descending coronary artery; LCX, Left Circumflex coronary artery; RCA, Right Coronary Artery; CRP, C - reactive protein; IL - 6, Interleukin - 6; PPAR - γ, Peroxisome Proliferator - Activated Receptor - gamma; TBR, Target - to - Background Ratio; hsCRP, High - sensitivity C - reactive protein; DN, Diabetic Nephropathy; UACR, Urinary Albumin - Creatinine Ratio; CCR2, C - C chemokine receptor type 2; ACR, Albumin: Creatinine Ratio; IL - 18, Interleukin - 18; AUC, Area Under the Curve; TGFα, Transforming Growth Factor - alpha; DR, Diabetic Retinopathy; BCVA, Best Corrected Visual Acuity; LFA - 1, Leukocyte Function - Associated Antigen - 1; DME, Diabetic Macular Edema; CMT, Central Macular Thickness; FDP, Frequency Doubling Perimetry.

The mammalian target of rapamycin (mTOR) pathway regulates the energy metabolism of immune cells. mTOR inhibitors suppress mTORC1 signaling, promoting the differentiation of Tregs (increasing the number of Foxp3+ cells), inhibiting the secretion of IL-17 by Th17 cells, preventing the polarization of macrophages into the M1 phenotype, and reducing the release of TNF-α and inducible nitric oxide synthase (iNOS) (139). In a study exploring the role of the mTOR pathway in fibroblast responses to TGF-β, it was demonstrated that TGF-β activates mTORC1 in fibroblasts via a PI3K-Akt-TSC2-dependent pathway, while the mTOR inhibitor rapamycin blocks TGF-β-mediated anchorage-independent growth without interfering with TGF-β-induced transcription or extracellular matrix production (140). Nonetheless, its clinical application is limited due to immunosuppressive side effects and potential effects on glucose metabolism. Currently, it is primarily utilized in experimental treatments or as part of combination therapy protocols.

Current drug interventions aim at the functional abnormalities of both innate and adaptive immune cells, exhibiting multiple effects such as anti-inflammation, anti-fibrosis, and promotion of repair in diabetic vascular complications. Future research should further integrate clinical trial data to clarify the precise regulatory effects of different drugs on specific immune cell subsets, providing a basis for personalized therapy.

### 6.2 Emerging therapies

#### 6.2.1 Stem cell therapies for diabetic vascular complications

Mesenchymal stem cells (MSCs) exhibit significant potential for repairing damaged tissues and preventing the progression of diabetic vascular complications (141, 142). MSCs have multi-directional differentiation potential, immunomodulatory effects, and paracrine functions, which allow them to play a positive role in the pathological process of diabetic vascular complications. In animal experiments and some clinical studies, MSCs can differentiate into vascular endothelial cells, smooth muscle cells, and so forth, promoting angiogenesis and enhancing the blood supply to damaged tissues (143–145). This has potential significance for the treatment of microvascular complications, such as diabetic retinopathy and diabetic nephropathy, as well as macrovascular complications, including peripheral arterial disease. For instance, studies have indicated that MSCs can stimulate the proliferation and migration of vascular endothelial cells and foster the formation of new blood vessels through the secretion of cytokines, including VEGF (146).

Simultaneously, the immunomodulatory function of MSCs can regulate the immune imbalance in diabetic patients and inhibit excessive inflammatory responses (141). In the context of diabetic vascular complications, inflammation is one of the key factors leading to tissue damage and disease progression. MSCs can suppress the secretion of pro-inflammatory cytokines, stimulate the production of anti-inflammatory cytokines, and modulate immune cell activity, thereby mitigating the damage inflammation inflicts on blood vessels and tissues. Furthermore, MSCs may also secrete various bioactive substances, such as exosomes, via paracrine mechanisms. These exosomes, which contain proteins and nucleic acids, can regulate cell growth, proliferation, and differentiation, and foster the repair of damaged tissues (147).

However, stem cell therapy also carries potential risks. First, immune rejection is a significant concern. Although MSCs have relatively low immunogenicity, immune reactions may still be triggered during allogeneic transplantation, leading to the rejection of transplanted cells and affecting the treatment’s efficacy. Second, the tumorigenic potential of stem cells is also a major worry. If stem cells experience abnormal proliferation or differentiation within the body, there is a risk of tumor formation, posing a serious threat to patients’ health. Moreover, standardized operating procedures for stem cell therapy have not been fully established, and the quality of stem cells from various sources and prepared through different methods varies significantly, introducing certain risks to clinical applications. However, in terms of benefits, stem cell therapy offers new approaches and methods for treating diabetic vascular complications. It is anticipated to surpass the constraints of conventional treatment approaches, achieving the regeneration and functional restoration of impaired tissues, thereby significantly enhancing the quality of life for patients. Furthermore, its immunomodulatory and tissue-repair capabilities could fundamentally delay or prevent the advancement of diabetic vascular complications, decrease the risk of severe complications in patients, and alleviate the medical burden.

#### 6.2.2 Precision medicine applications based on immune biomarkers

Precision medicine strategies for diabetic vascular complications incorporate patients’ immune profiles, genetic backgrounds, and clinical phenotypes to facilitate personalized treatment. The stratification based on immune biomarkers significantly enhances therapeutic efficacy and decreases adverse reactions. For instance, the Th1/Th2 immune balance is instrumental in guiding risk assessment for complications: patients with a Th1-predominant profile are more susceptible to microvascular complications, whereas those with a Th2-dominant profile may have a greater risk of macrovascular disease, necessitating targeted interventions with IL-12 inhibitors or IL-4/IL-13 agonists (148). In a case-control study examining T cell subset balance in type 2 diabetic patients, researchers found that individuals with diabetic nephropathy exhibited significantly higher Th17 cell counts and Th17/Treg ratios, alongside lower Treg cell counts, compared to diabetic patients without nephropathy and healthy controls. This imbalance highlights the pro-inflammatory dominance of Th17 cells and the attenuation of Treg-mediated immunosuppression in diabetic nephropathy progression (149). Clinically, such a low Treg/Th17 ratio suggests heightened susceptibility to Th17-driven renal injury, implying potential responsiveness to IL-17 antagonists that block pro-inflammatory signaling. Conversely, a relatively higher ratio, characterized by robust Treg activity, may indicate greater benefit from Treg activation therapies aimed at restoring immune tolerance and reducing glomerular inflammation. These findings establish the Treg/Th17 ratio as a predictive biomarker for personalized therapeutic strategies in diabetic nephropathy, tailoring interventions to either dampen Th17-mediated inflammation or enhance Treg protective functions based on individual immune profiles.

Biomarker-driven drug selection further optimizes outcomes. Patients with diabetic retinopathy and elevated sTNFR1/sTNFR2 levels exhibit a higher response rate to TNF-α inhibitors, such as infliximab (150). Conversely, those with high urinary miR-200c levels experience delayed renal decline when treated with TGF-β inhibitors. Real-time immune monitoring through omics technologies enables dynamic treatment adjustments. Despite challenges related to standardization, cost, and safety, immune-guided precision medicine presents innovative opportunities for the personalized management of diabetic vascular complications by integrating biomarker insights with therapeutic decisions.

## 7 Challenges and future directions

Currently, although progress has been made in research on the relationship between diabetic vascular complications and immune cells, there are still numerous knowledge gaps and areas that require further exploration. In basic research, the mechanisms behind the dynamic changes of immune cells during the onset and progression of diabetic vascular complications are not fully understood. The intricate interactions among various immune cell subsets and their functional alterations at different stages of the disease remain to be investigated in depth. For instance, in the context of diabetic nephropathy, the cooperative mechanisms among macrophages, T cells, and B cells remain unclear. Additionally, the functional transitions of these cells throughout the early and late stages of the disease have not been thoroughly investigated. Furthermore, the mechanism behind the interaction between the gut microbiota and immune cells in diabetic vascular complications, as well as the influence of genetic and environmental factors on this process, necessitates further research.

From a clinical perspective, existing treatment methods still have limitations. Although some drugs targeting immune cell receptors have theoretical therapeutic potential, their long-term safety and efficacy have not been fully substantiated in clinical trials. Some drugs may cause adverse reactions. For instance, immunosuppressants may elevate the risk of infection, impacting the overall health of patients. Simultaneously, the application of precision medicine in treating diabetic vascular complications remains in the exploratory phase. While the stratification treatment and drug selection based on immune biomarkers hold promise, accurately applying these biomarkers in clinical practice and developing standardized detection and treatment protocols is a significant challenge. Additionally, there is limited research on gender differences related to immune cells in diabetic vascular complications. Differences in immune responses and disease progression between male and female patients may exist. Future research should focus more on this area to achieve more precise personalized treatment.

Looking ahead, in-depth research into these knowledge-gap areas is crucial for uncovering the pathogenesis of diabetic vascular complications and developing more effective treatment strategies. On the one hand, strengthening basic research and delving deeply into the functions of immune cells, their interactions, and their associations with other factors will provide a theoretical foundation for the development of new therapeutic targets. On the other hand, accelerating the pace of clinical research, verifying the effectiveness and safety of existing treatment methods, and promoting the development of precision medicine to develop personalized treatment plans according to patients’ individual characteristics are expected to significantly improve the prognosis of diabetic patients and reduce the burden of diabetic vascular complications. Meanwhile, interdisciplinary research will be an important future development direction. Integrating the knowledge of immunology, endocrinology, genetics, microbiology, and other disciplines to comprehensively and deeply understand the pathogenesis of diabetic vascular complications will provide new ideas and methods for solving this problem.
